# Systemic but not MDSC-specific IRF4 deficiency promotes an immunosuppressed tumor microenvironment in a murine pancreatic cancer model

**DOI:** 10.1007/s00262-020-02605-9

**Published:** 2020-05-24

**Authors:** Philipp Metzger, Sabrina V. Kirchleitner, Daniel F. R. Boehmer, Christine Hörth, Angelika Eisele, Steffen Ormanns, Matthias Gunzer, Maciej Lech, Kirsten Lauber, Stefan Endres, Peter Duewell, Max Schnurr, Lars M. König

**Affiliations:** 1 Center of Integrated Protein Science Munich (CIPSM) and Division of Clinical Pharmacology, University Hospital, LMU Munich, Munich, Germany; 2 Department of Radiation Oncology, University Hospital, LMU Munich, Munich, Germany; 3 Department of Neurosurgery, University Hospital, LMU Munich, Munich, Germany; 4 Institute for Medical Information Processing, Biometry, and Epidemiology - IBE, Chair of Public Health and Health Services Research, LMU Munich, Munich, Germany; 5 Pettenkofer School of Public Health, Munich, Germany; 6 Institute of Pathology, Faculty of Medicine, LMU Munich, Munich, Germany; 7 Institute for Experimental Immunology and Imaging, University Hospital, University Duisburg- Essen, Essen, Germany; 8 Leibniz Institute for Analytical Sciences - ISAS, Dortmund, Germany; 9 Department of Medicine IV, University Hospital, LMU Munich, Munich, Germany; 10 German Center for Translational Cancer Research (DKTK), Partner Site, Munich, Germany; 11 Einheit für Klinische Pharmakologie (EKLiP), Helmholtz Zentrum München, German Research Center for Environmental Health (HMGU), Neuherberg, Germany; 12 Institute of Innate Immunity, University of Bonn, Bonn, Germany

**Keywords:** Myeloid-derived suppressor cells (MDSC), IRF4, Immunosuppression, Pancreatic cancer, Myelopoiesis

## Abstract

Pancreatic ductal adenocarcinoma is characterized by a strong immunosuppressive network with a dense infiltration of myeloid cells including myeloid-derived suppressor cells (MDSC). Two distinct populations of MDSC have been defined: polymorphonuclear MDSC (PMN-MDSC) and monocytic MDSC (M-MDSC). Several factors influence the development and function of MDSC including the transcription factor interferon regulatory factor 4 (IRF4). Here, we show that IRF4 deficiency accelerates tumor growth and reduces survival, accompanied with a dense tumor infiltration with PMN-MDSC and reduced numbers of CD8^+^ T cells. As IRF4 has been described to modulate myeloid cell development and function, particularly of PMN-MDSC, we analyzed its role using MDSC-specific IRF4 knockout mice with the Ly6G or LysM knock-in allele expressing *Cre recombinase* and *Irf4*^*flox*^. In GM-CSF-driven bone marrow cultures, IRF4 deficiency increased the frequency of MDSC-like cells with a strong T cell suppressive capacity. Myeloid (LysM)-specific depletion of IRF4 led to increased tumor weight and a moderate splenic M-MDSC expansion in tumor-bearing mice. PMN cell (Ly6G)-specific depletion of IRF4, however, did not influence tumor progression or MDSC accumulation in vivo in accordance with our finding that IRF4 is not expressed in PMN-MDSC. This study demonstrates a critical role of IRF4 in the generation of an immunosuppressive tumor microenvironment in pancreatic cancer, which is independent of IRF4 expression in PMN-MDSC.

**Electronic supplementary material:**

The online version of this article (10.1007/s00262-020-02605-9) contains supplementary material, which is available to authorized users.

## Introduction

The tumor microenvironment (TME) contains a variety of immune cells with opposing functions. While anti-tumoral cells such as cytotoxic T cells, T helper cells and natural killer cells are important in the immune surveillance and protection against tumors, certain immune modulatory cells including myeloid subsets shape the TME during immune equilibrium and escape phase [1] and enhance tumor progression by dampening the effector cells [2]. Three main subsets within the myeloid compartment are characterized by a strong T cell suppressive capacity. Depending on the cell of origin and marker expression, they can be classified in (1) monocytic myeloid-derived suppressor cells (M-MDSC), (2) polymorphonuclear myeloid-derived suppressor cells (PMN-MDSC) and (3) tumor-associated macrophages (TAM) [3, 4]. Under pathological activation such as chronic inflammation or cancer MDSC are expanded and gain a suppressive phenotype [5]. Increased numbers of MDSC are a negative prognostic factor for the survival of patients with pancreatic cancer (PDAC) [6, 7]. In addition, a MDSC-enriched TME is a negative predictive marker for the response to immunotherapy in a mouse model of PDAC [8]. Despite their high clinical relevance, MDSC-targeted treatment approaches are still insufficient [9] and there is great need for a better understanding of MDSC development and activation in cancer [10].

Both MDSC subsets develop from the granulocyte macrophage precursor (GMP) in the bone marrow, and several transcription factors are associated with development and function of MDSC [11]. Signal transducer and activator of transcription 3 (STAT3) has been shown to regulate MDSC expansion by mediating myeloid-specific growth factor signaling [12] as well as the suppressive activity by inducing Arginase 1 (ARG1) expression, which suppresses T cell activity by degrading the essential amino acid arginine [13]. Expression of arginase is one key suppressive mechanism of MDSC.[3]. CCAAT/enhancer-binding protein beta (*C/EBPβ*) regulates the development of polymorphonuclear cells under inflammatory conditions and has been also associated with PMN-MDSC development [14]. The transcription factor interferon regulatory factor 8 (IRF8) plays a central role in the development of monocytic and polymorphonuclear cells from GMP [15]. IRF8-deficient mice show leukemic-like symptoms with a dense accumulation of polymorphonuclear cells [16]. In the context of cancer, strong accumulation of PMN-MDSC has been reported for IRF8-deficient mice [17].

Another transcription factor of the IRF family, IRF4 is known for its function in lymphoid cells. There, IRF4 regulates antibody class switching in B cells [18, 19] and facilitates the differentiation of naïve CD4^+^ T cells into T helper cell subsets such as Th2, Th9 or Th17 [20]; in CD8^+^ T cells, IRF4 plays a crucial role in maintaining effector function [21, 22]. Besides its importance in the lymphoid lineage, IRF4 expression has been shown to regulate similar biological functions as observed for IRF8 in myeloid cells. IRF4 is expressed in myeloid-precursor cells and shifts the myeloid development from neutrophil to monocyte development, but to a lesser extent than IRF8 [23]. In contrast to early myeloid cell development, IRF4 and IRF8 play opposing roles in the development of conventional dendritic cells (cDC) that develop from GMP via pre-cDC precursor cells. While IRF4 is one of the lineage-defining transcription factors in the differentiation of cDC2, IRF8 is pivotal for the differentiation of cDC1 [24, 25]. Recently, it has been shown that differentiation of Ly6C^+^ monocytes to monocytic DC (moDC) requires IRF4 [26]. Moreover, IRF4 expression is induced by cytokines mediating M2 alternatively activated macrophage polarization such as IL-4 and IL-13 [27], and itself regulates M2 polarization by inducing M2 effector genes like Arg1 [28]. A recent publication indicates that IRF4 may regulate the differentiation and suppressive function of MDSC [29].

In this study, we aim at understanding the role of IRF4 in MDSC development and function in a murine model of PDAC which is characterized by strong expansion of MDSC [30, 31], by using mice with either global, myeloid cell- or granulocyte-specific *Irf4* deletion.

## Results

### Systemic IRF4 deficiency accelerates PDAC tumor growth and expands MDSC in vivo

**Fig. 1:**
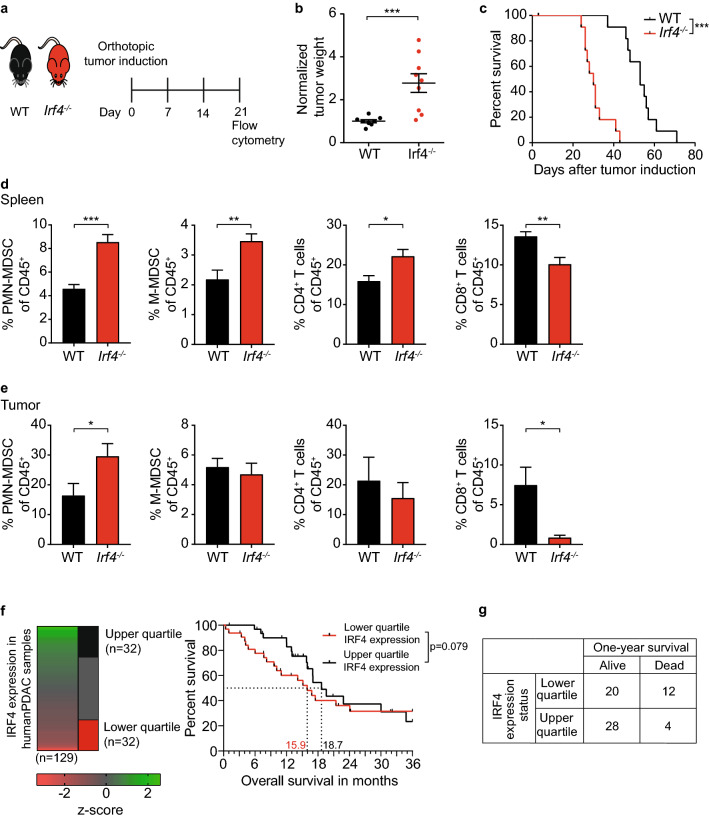
IRF4 deficiency accelerates PDAC tumor growth and expansion of MDSC populations in blood and spleen. **a**–**e** T110299 tumors were induced orthotopically in *Irf4*^−/−^ and wild-type (WT) mice. After 21 days, tumor weight was measured (**b**). Kaplan–Meier curve depicts survival of orthotopic PDAC-bearing mice (**c**). Immune cell frequencies in spleen (**d**) and tumor (**e**) were analyzed by flow cytometry. Differences between genotypes were statically analyzed using the Mann–Whitney U test (**b**, **d**, **e**) or Log rank test (**c**). **a**–**e** Pooled data from 2–3 independent experiments are shown, error bars represent mean ± SEM (*n* = 3–8 mice/group). **e** IRF4 expression level of metastasized PDAC patients and Kaplan–Meier survival curve of patients from the upper and lower quartile of IRF4 expression is displayed for 36 months. Median survival of both groups in months is indicated with dotted lines (**f**). Contingency table of patients based on their survival status after one year and IRF4 expression level (**g**). Asterisks indicate *p < 0.05, **p < 0.01, ***p < 0.001

To study the role of IRF4 in pancreatic cancer we made use of a transplantable, orthotopic tumor model using the tumor cell line T110299, which originate from a genetically engineered, spontaneous mouse model with PDAC-characteristic driver mutations (*Kras*^*G12D*^* Tp53*^*R172H*^). We recently reported that this model is particularly enriched for myeloid cells such as MDSC [31]. Three weeks after orthotopic tumor induction, tumor weight of *Irf4*^*−/−*^ mice was significantly increased compared to IRF4 sufficient *Irf4*^*flox/flox*^ controls (Fig. 1a, b). Moreover, the survival of tumor-bearing *Irf4*^*−/−*^ mice was significantly reduced (Fig. 1c). We noted no significant differences in histologic tumor morphology between both genotypes (Fig. S1). T110299 tumors from both control and *Irf4*^*−/−*^ mice showed a moderately differentiated tubular to tubulo-papillary adenocarcinoma pattern with similar amounts of desmoplastic stroma and moderate chronic inflammatory infiltrates, resembling human pancreatic ductal adenocarcinoma. To study the immunological consequences of IRF4 deficiency, immune cell frequency of T110299 tumor-bearing mice was analyzed three weeks after tumor induction by flow cytometry. In the spleen of *Irf4*^*−/−*^ mice, PMN-MDSC, M-MDSC and CD4^+^ T cell frequencies were significantly increased, whereas the frequency of CD8^+^ T cells was reduced (Fig. 1d). In the TME, PMN-MDSC frequency was significantly increased in *Irf4*^*−/−*^ mice, whereas the frequency of CD8^+^ T cells was dramatically reduced, indicating a profound immunosuppressive TME in mice deficient for IRF4 (Fig. 1e) that may account for the more rapid tumor progression in these mice.

### Low IRF4 expression in PDAC patients is associated with reduced one-year survival

To confirm these findings in the human disease, the effect of IRF4 expression on the survival of metastasized PDAC patients was analyzed using the Cancer Genome Atlas (TCGA) data set. Metastasized PDAC patients were categorized based on their IRF4 expression level in two groups (upper and lower quartile) and survival of these selected groups was analyzed. The median survival of patients with low IRF4 expression was 15.9 months versus 18.7 months of patients with high expression levels. There was no significant difference in the overall survival in regard to the IRF4 expression (Fig. 1f). However, *χ*^2^-test of survival after 1 year revealed a significantly reduced survival of patients with lower IRF4 expression (*χ*^2^ = 5.33, *p* = 0.02) (Fig. 1g).

### IRF4 is expressed in M-MDSC, but not PMN-MDSC

**Fig. 2:**
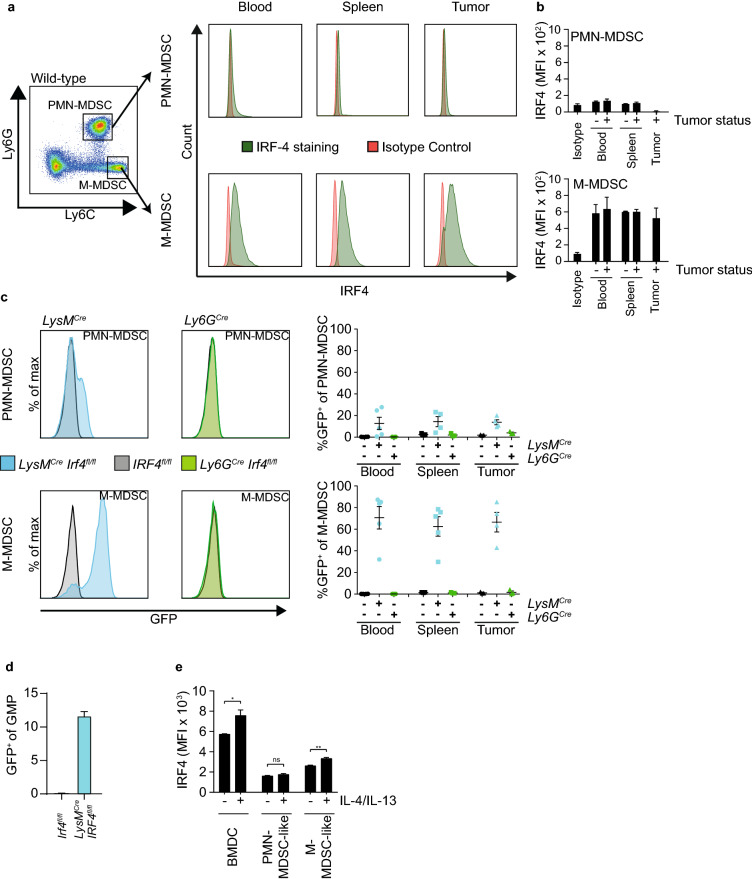
IRF4 is expressed in monocytic but not polymorphonuclear cells. **a**–**d** KPC-derived T110299 tumors were induced orthotopically. **a**, **b** IRF4 expression of MDSC subsets from tumor-free and PDAC-bearing wild-type mice was analyzed by flow cytometry. **a** A representative histogram of anti-IRF4 staining as well as isotype control staining from a PDAC-bearing mouse is depicted. After 21 days, GFP expression of MDSC (**c**) and GMP (**d**) subsets of *Ly6G*^*Cre*^*Irf4*^*fl/fl*^ (green), *LysM*^*Cre*^*Irf4*^*fl/fl*^ (blue) and *Irf4*^*fl/fl*^ (black) mice was determined by FACS analysis. **e** IRF4 expression of myeloid cells from bone marrow cultures were analyzed by flow cytometry in the absence and presence of IL-4 and IL-13. The difference between stimulated and unstimulated cells was statistically analyzed using unpaired student’s t-test. Error bars represent mean ± SEM (*n* = 2–5 mice/group), asterisks indicate *p < 0.05, **p < 0.01

To better understand the role of IRF4 in the initiation of the immunosuppressive and myeloid cell-enriched TME, IRF4 expression of both monocytic and polymorphonuclear cells in tumor-free and T110299 tumor-bearing mice was analyzed by flow cytometry. IRF4 was expressed homogenously in monocytic cells in blood, spleen as well as tumor, and was independent of the tumor status. In contrast, IRF4 expression in polymorphonuclear cells in blood, spleen and tumor was absent (Fig. 2a, b).

As this is in contrast to published data [29], we further validated this finding by alternative approaches and used conditional myeloid-specific IRF4 knockout mouse models. *Irf4*^flox^ mice have been designed to express green fluorescent protein (GFP) instead of IRF4 upon successful *Cre*-mediated recombination under the same physiological control as IRF4 [18]. Therefore, the conditional IRF4 knockout mice can also act as reporter mice. *LysM*^*Cre*^*Irf4*^*fl/fl*^ mice have an IRF4 deletion in polymorphonuclear cells, monocytes, macrophages and partly dendritic cells [32], whereas *Ly6G*^*Cre*^*Irf4*^*fl/fl*^ mice have the IRF4 deletion in polymorphonuclear cells only [33].

In line with our previous observation, M-MDSC of the *LysM*^*Cre*^*Irf4*^*fl/fl*^ mice were positive for GFP in the blood, spleen and tumor. No GFP expression was detectable in PMN-MDSC or M-MDSC of the *Ly6G*^*Cre*^*Irf4*^*fl/fl*^ mice. In contrast, in *LysM*^*Cre*^*Irf4*^*fl/fl*^ mice, a small fraction of approximately 15% of GFP^+^ PMN-MDSC was detectable in blood, spleen and tumor (Fig. 2c). As the GFP expression in those PMN-MDSC may derive from its precursor cell expressing LysM, we characterized GFP expression of GMP in *LysM*^*Cre*^*Irf4*^*fl/fl*^ mice. Indeed, 12% of GMP expressed GFP indicating that IRF4 is expressed early in the myeloid cell development (Fig. 2d). To further characterize the cell-specific expression pattern of IRF4 in myeloid cells, bone marrow cells were stimulated overnight with the known IRF4 inducers IL-4 and IL-13. As expected, CD11b^+^ CD11c^+^ MHC-II^+^ bone marrow-derived dendritic cells (BMDC) responded to cytokine stimulation by upregulating IRF4 expression. IRF4 expression was only inducible in CD11b^+^Ly6C^high^Ly6G^−^ M-MDSC-like cells, but not in CD11b^+^Ly6C^int^Ly6G^+^ PMN-MDSC-like cells (Fig. 2e). Taken together, the data demonstrate that IRF4 is expressed in M-MDSC and myeloid precursor cells but is absent in mature Ly6G-expressing PMN-MDSC.

### IRF4 deficiency expands suppressive MDSC-like cells in vitro

**Fig. 3:**
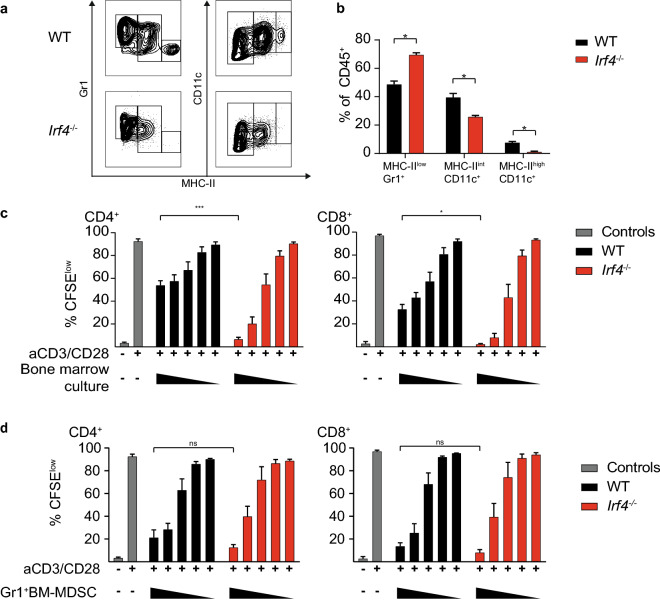
IRF4 deficiency leads to MDSC-like cell expansion in bone marrow cultures in vitro. **a**–**d** Bone marrow cells from WT and *Irf4*^−/−^ mice were cultured for seven days in the presence of GM-CSF. **a**, **b** Cell composition of bone marrow cultures was measured. **c** Bone marrow cells from WT and IRF4-deficient mice were co-cultured with CFSE-labeled T cells and proportion of proliferated CFSE^low^ CD8^+^ and CD4^+^ T cells was analyzed by flow cytometry. **d** Gr1high MHC-IIlow cell population of WT and IRF4-deficient bone marrow cultures were FACSorted, co-cultured with CFSE-labeled T cells and the proportion of proliferated CFSE^low^ CD8^+^ and CD4^+  ^was determined. The difference between genotypes was statistically analyzed using Mann–Whitney U test, error bars represent mean ± SEM (*n* = 3), asterisks indicate *p < 0.05 and ***p < 0.001

GM-CSF-driven bone marrow cultures are an established model to study MDSC in vitro [14]. To evaluate the role of IRF4 in the development and function of MDSC, bone marrow cells of wild-type and *Irf4*^*−/−*^ mice were cultured for seven days in the presence of GM-CSF, and cell composition was analyzed. The frequency of MDSC-like (Gr1^+^MHC-II^low^) cells was significantly increased in bone marrow cultures from *Irf4*^*−/−*^ mice, while both MHC-II^int^ and MHC-II^high^ BMDC were significantly reduced (Fig. 3a, b). Next, T cell suppressive capacity of bone marrow-derived MDSC was measured in T cell co-cultures in the presence of anti-CD3/anti-CD28 beads. T cell proliferation was measured and revealed that IRF4-deficient bone marrow culture cells suppressed the proliferation of both CD4^+^ and CD8^+^ T cells more potently, as compared to wild-type controls (Fig. 3c). However, as the difference in MDSC composition may influence the overall suppressive activity, FACS-sorted Gr1^high^MHC-II^low^ MDSC-like cells from wild-type and *Irf4*^*−/−*^ mice were individually analyzed in a T cell suppression assay. Sorted Gr1^+^ MDSC-like cells of wild-type and *Irf4*^*−/−*^ mice exhibited similar T cell suppressive capacities, arguing against a direct role of IRF4 in controlling the suppressive capacity of MDSC-like cells (Fig. 3d).

### Myeloid-specific deletion of IRF4 accelerates PDAC growth and expands M-MDSC numbers

**Fig. 4:**
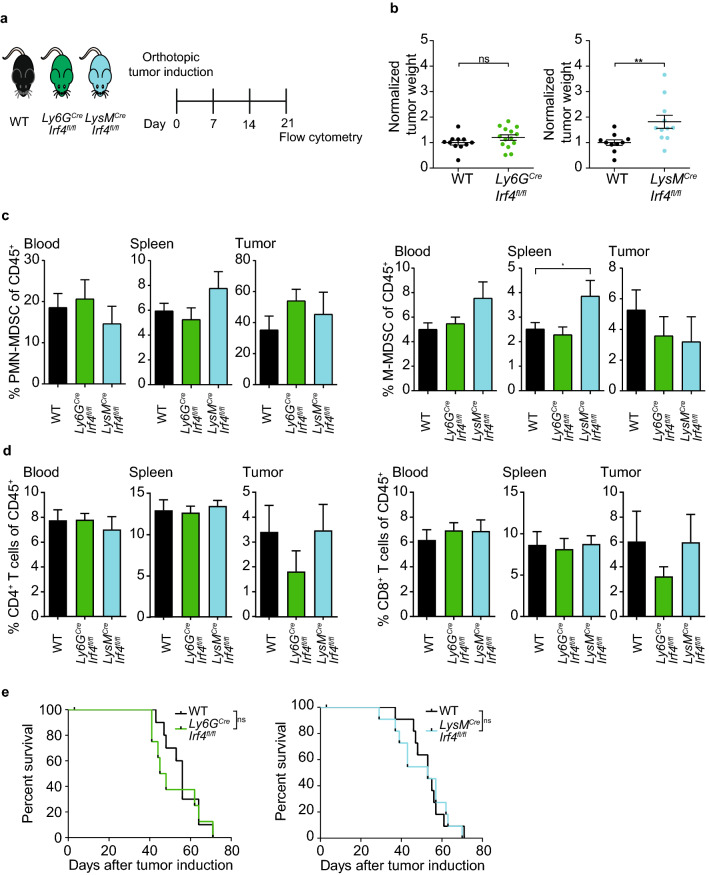
Targeted deletion of IRF4 in *LysM*^*Cre*^ but not *Ly6G*^*Cre*^ mice increases tumor growth without influencing survival. **a**–**d** KPC-derived T110299 tumors were induced orthotopically in *Ly6G*^*Cre*^*Irf4*^*fl/fl*^ (green), *LysM*^*Cre*^*Irf4*^*fl/fl*^ (blue) and *Irf4*^*fl/fl*^ (black) mice. After 21 days, **a**, **b** tumor weight was measured and **c** MDSC as well as **d** T cell frequency was analyzed by flow cytometry. **e** Survival of PDAC-bearing mice was monitored. Statistical analysis was performed using Kruskal Wallis test, followed by Dunn’s multiple comparison test between controls and conditional knockout mice. Pooled data from three independent experiments are depicted, error bars represent mean ± SEM (*n* = 8–14 mice/group), *p* value of Dunn’s test are shown in the graph, asterisks indicate *p < 0.05 and **p < 0.01

The two conditional IRF4 knockout mouse models described above were used to study the intrinsic role of IRF4 in myeloid cells in vivo. KPC-derived T110299 tumors were orthotopically induced in *Ly6G*^*Cre*^*Irf4*^*fl/fl*^ mice, *LysM*^*Cre*^*Irf4*^*fl/fl*^ mice as well as *Irf4*^*fl/fl*^ control mice (Fig. 4a). While there was no significant difference in tumor growth between *Ly6G*^*Cre*^*Irf4*^*fl/fl*^ and control mice, tumor weight was significantly increased in *LysM*^*Cre*^*Irf4*^*fl/fl*^ mice, as compared to *Irf4*^fl/fl^ control mice (Fig. 4b). Flow cytometric analysis of MDSC frequencies three weeks after tumor induction showed no difference in PMN-MDSC frequency in blood, spleen and tumor of *LysM*^*Cre*^*Irf4*^*fl/fl*^ mice and *Ly6G*^*Cre*^*Irf4*^*fl/fl*^ mice compared to *Irf4*^*fl/fl*^ controls; however, M-MDSC frequency was moderately increased in the spleen of *LysM*^*Cre*^*Irf4*^*fl/fl*^ mice (Fig. 4c). Compared to *Irf4*^*fl/fl*^ controls, there was no significant difference in CD4^+^ or CD8^+^ T cell frequencies in *LysM*^*Cre*^*Irf4*^*fl/fl*^ mice or *Ly6G*^*Cre*^*Irf4*^*fl/fl*^ mice (Fig. 4d). Of note, both myeloid-specific IRF4-deficiencies had no influence on the survival of PDAC-bearing mice (Fig. 4e).

In conclusion, this study demonstrates that only early myeloid precursor cells and M-MDSC express IRF4, whereas mature PMN-MDSC lack IRF4 expression. In our hands, specific deletion of IRF4 in PMN-MDSC has no influence on PMN-MDSC expansion, their T cell suppressive capacity, tumor growth or survival. In contrast, myeloid cell-specific deletion of IRF4 in *LysM*^*Cre*^*Irf4*^*fl/fl*^ mice slightly accelerates tumor growth, which was accompanied by increased M-MDSC frequency in the spleen. No impact on PMN-MDSC numbers or survival is seen in these mice. These findings argue against a pro-tumoral effect of IRF4 in PMN-MDSC. Hence, as observed in mice with global IRF4 deficiency, the IRF4-mediated effects in other immune cells likely account for the immunosuppressive TME with its dense MDSC infiltration.

## Discussion

In this study, we show that global IRF4 deficiency accelerates tumor growth, increases frequencies of tumor-infiltrating PMN-MDSC and reduces survival in a murine PDAC model. This is in line with human TCGA cohort data showing that the one-year survival of metastasized PDAC patients with low IRF4 expression is significantly reduced. Reporter mice for studying IRF4 expression in myeloid cells in vivo revealed the induction of IRF4 in myeloid precursor cells and M-MDSC, but not in PMN-MDSC. Accordingly, IRF4 deletion in Ly6G^+^ cells had no influence on MDSC frequency, the suppressive function, tumor growth or survival. In contrast, IRF4 deletion in LysM^+^ cells increased tumor weight and led to moderately expanded M-MDSC population in the spleen; yet there was no impact on survival.

A recent study reported that IRF4 is expressed by PMN-MDSC as well as M-MDSC and that 4T1 breast tumors reduced the expression of IRF4 in both cell types. Furthermore, the study demonstrated by knock-down and overexpression experiments in bone marrow cultures showed that lower IRF4 expression increased the frequency of PMN-MDSC and was associated with higher suppressive activity of MDSC. A myeloid cell-specific knockout of IRF4 increased the tumor weight in B16F10 tumor bearing mice [29]. In contrast to this report, we clearly demonstrate by the use of reporter mice as well as antibody staining that in our PDAC model IRF4 expression is limited to M-MDSC, whereas PMN-MDSC lack IRF4 expression [29]. As the 4T1 cell line was generated from a BALB/c mouse and the PDAC model we used originated from a C57BL/6 mouse, it remains open whether this discrepancy is due to different tumor models or mouse strains used.

A fraction of GMP was found to express IRF4, which is why they likely contain precursors for both M-MDSC and PMN-MDSC. In line with this finding, a small proportion of PMN-MDSC exhibited a weak GFP expression in *LysM*^*Cre*^*Irf4*^*flox*^ reporter mice. Taking together, the findings argue that IRF4 is expressed in some precursors of granulocytes, but is lost during PMN-MDSC differentiation. Considering our data and the current literature of IRF4 and IRF8 data on early myeloid cell development, IRF4 and IRF8 have seemingly overlapping functions in myeloid cell development. The data also suggest that IRF8 may compensate for IRF4 function in IRF4-deficient animals [23]; however, the exact molecular mechanism of how IRF4-regulates cell fate decision remains elusive.

The suppressive function of MDSC has been linked to their immature state [34]. The GM-CSF-driven bone marrow culture system is frequently used to study dendritic cells and MDSC in vitro [14, 29]. IRF4-deficiency shifts the bone marrow culture from mature dendritic cells towards immature MDSC-like cells. This shift in the population frequency questions again earlier findings showing that induction of IRF4 decreases and inhibition of IRF4 increases the suppressive capacity of MDSC intrinsically [29]. Using sorted MDSC-like cells from these cultures as well as genetic deletion instead of siRNA knock-down, we demonstrate that the intrinsic suppressive activity is not directly influenced by IRF4.

Given the result that IRF4 is not expressed in polymorphonuclear cells, it was not surprising that the deletion of IRF4 in Ly6G^+^ cells in vivo did not influence tumor growth, overall survival or MDSC cell frequency. In line with the previous report [29], myeloid-specific deletion of IRF4 using the *LysM*^*Cre*^*Irf4*^*fl/fl*^ mice, accelerated tumor growth, but had no influence on the overall survival. The frequency of M-MDSC was moderately elevated in the spleen, but not in tumors, suggesting that IRF4 rather impacts monocyte differentiation or proliferation.

Besides the minor effects of myeloid-specific deletion of IRF4, the accelerated tumor growth in global IRF4-deficient mice demonstrates a central role of IRF4 in shaping the TME and anti-tumor immunity. IRF4 has been described to be pivotal for efficient antigen cross-presentation of moDC [26] and to be required for sustained CD8^+^ T cell activation [21, 22]. Given the substantial reduction of intratumoral CD8^+^ T cells, it seems more likely that this is due to an impaired sustained activation of antitumoral T cells than due to the amplification and action of MDSC in IRF4-deficent mice. We recently demonstrated that in the PDAC model used the PMN-MDSC frequency correlates with tumor weight [31]. One can therefore argue that the elevated levels of PMN-MDSC in the global IRF4 deficient mouse could be a secondary effect of the increased tumor size.

In summary, we demonstrate that IRF4 plays an important role in shaping the immune cell composition in the TME of murine pancreatic cancer. Due to the increased PMN- and M-MDSC frequency in IRF4 knockout mice, we hypothesized that a MDSC-intrinsic role of IRF4 might explain the effect on tumor progression. However, polymorphonuclear cells do not express IRF4 in tumor bearing mice and in line with that, the deletion of IRF4 in Ly6G^+^ cells did not alter tumor development. Despite some effects of IRF4 deletion in LysM^+^ cells on tumor growth, again, no effect on survival or PMN-MDSC accumulation was observed. Our results, therefore, suggest that the observed in vivo effects in globally IRF4-deficient mice are secondary and due to globally imbalanced immune regulation, but not due to an IRF4-intrinsic effect in MDSC.

## Material and methods

### Mice

C57BL/6 wild-type mice were purchased from Janvier, France. *Irf4*^*flox*^ mice (B6.129S1-*Irf4*^*tm1Rdf*^/J) were a kind gift from Prof. Bopp (Institute of Immunology, Universitätsmedizin Mainz), *Ly6G*^*Cre*^ mice (C57BL/6-*Ly6g*^(tm2621(Cre−tdTomato)^Arte)) were a kind gift from Prof. Gunzer (Institute for Experimental Immunology and Imaging, University of Duisburg-Essen), *LysM*^*Cre*^ mice (B6.129P2-*Lyz2*^*tm1(cre)Ifo*^/J) were a kind gift from PD Dr. Lech (Institute of Clinical Biochemistry, Klinikum der Universität München). *FLP1* recombinase expressing FLPe mice (B6;SJL-Tg^(ACTFLPe)9205Dym)^/J) were purchased from Jackson Laboratory (Sulzfeld, Germany). All mice were kept on C57BL/6 background with a 12-h light/dark cycle, water *ad lib*. and regular chow diet (sniff, Soest, Germany) at the Klinikum der Universität München, Munich, Germany. Experiments were performed according to national ethical guidelines approved by the local government (Regierung von Oberbayern, Munich, Germany; file number 55.2-1-54-2532-175-12). *LysM*^*Cre*^ were cross-bred with *Irf4*^*flox*^ mice to obtain *LysM*^*Cre*^*Irf4*^*flox*^*,* and *Ly6G*^*Cre*^ were cross-bred with *Irf4*^*flox*^ mice to obtain *Ly6G*^*Cre*^*Irf4*^*flox*^. Both mouse strains were kept on homozygous *Irf4*^*fl/fl*^* background*. Exons 1 and 2 of *Irf4* in B6.129S1-*Irf4*^*tm1Rdf*^/J mice are flanked by two FRT sites. To generate global IRF4-deficient mice, B6;SJL-Tg^(ACTFLPe)9205Dym^/J mice were cross-bred with B6.129S1-*Irf4*^*tm1Rdf*^/J mice and, as described before, *Irf4*^*−/−*^ mice were obtained [18]. IRF4 sufficient mice originating from those breedings were used as littermate controls. Genotypes of all mice were routinely analyzed by PCR.

### DNA isolation and genotyping

DNA from ear or tail biopsies was extracted and analyzed as described before [35]. Briefly, biopsies were incubated in 75 µl alkaline lysis buffer (25 mM NaOH, 0.2 mM EDTA in H_2_O) for 30 min at 95 °C and reaction was stopped by adding 75 µl neutralization buffer (40 mM Tris-HCl in H_2_O). Supernatant containing genomic DNA was subsequently used for genotyping with locus specific primer pairs listed in supplementary table 1 by using genotype-specific cycling programs, as summarized in supplementary table 2.

### Cell culture

Primary cells were cultured in RPMI-1640 medium (Sigma-Aldrich, Taufkirchen, Germany), supplemented with 10% fetal calf serum (FCS), 2 mM l-glutamine, 100 U/l penicillin, 0.1 mg/ml streptomycin, 100 mM non-essential amino acids (all gibco®, Thermo Fisher Scientific, Karlsruhe, Germany), 1 mM sodium pyruvate and 50 mM 2-mercaptoethanol (both Sigma Aldrich). T110299 tumor cells had been isolated from tumors of genetically-engineered *Ptf1a-Cre Kras*^*G12D*^* p53*^*fl/R172H*^ (KPC) mice and kindly provided by Prof. Siveke (West German Cancer Center (WTZ), University Hospital Essen). T110299 cells were cultured in DMEM high glucose media (Sigma-Aldrich), supplemented with 10% FCS, 2 mM l -glutamine, 100 U/l penicillin and 0.1 mg/ml streptomycin (all gibco®). All cells were kept in a humidified incubator at 37 °C and 5% CO_2_ and were regularly tested for mycoplasma contamination.

### Bone marrow culture

Bone marrow cells were isolated by flushing femur and tibia. 2 × 10^6^ cells per 10 ml were seeded in a 10 cm cell culture round plate in primary cell medium supplemented with 20 ng/ml GM-CSF (Peprotech, London, United Kingdom). After three days, 10 ml primary cell medium supplemented with 20 ng/ml GM-CSF was added. Five days after cell isolation, 66% of the medium containing 20 ng/ml GM-CSF was exchanged. If indicated, cells were stimulated overnight with 10 ng/ml IL-4 and 10 ng/ml IL-13 (both Peprotech, London, United Kingdom).

### Orthotopic tumor induction

Orthotopic tumors were induced by surgical implantation, as described before [36]. Briefly, 6–12 weeks old mice were anesthetized, and by surgical incision of the skin and peritoneum, the pancreas was carefully mobilized. After the injection of 2 × 10^5^ T110299 cells in 25 µl PBS, the pancreas was relocated, and the incision was closed by surgical suture. Mice were monitored daily and distressed mice were sacrificed. Tumor weight of sacrificed animals was measured and normalized to average tumor weight of WT animals in the respective experiment.

### Cell isolation

Spleens and tumors were isolated from the mice and blood was drawn. Spleens were processed through a 70 µm cell strainer. Erythrocytes from spleen and blood were removed using the red blood cell lysis buffer (BD Biosciences, Heidelberg, Germany). Tumor tissue was minced into pieces and mechanically dissociated using the mouse Tumor Dissociation Kit with the gentleMACS™ Dissociator application (both Miltenyi Biotech, Bergisch Gladbach, Germany), according to the manufacturer´s instructions. The cell suspension was separated from tissue debris by sequentially using 100 µm and 70 µm cell strainers. For functional assays, untouched T cells were isolated using the Pan T cell isolation Kit II, and for MDSC isolation the Myeloid-Derived Suppressor Cell Kit was used (both Miltenyi Biotec). In brief, in a two-step separation process, PMN-MDSC (CD11b^+^ Ly6C^int^ Ly6G^+^) were isolated with anti-Ly6G beads followed by M-MDSC (CD11b^+^ Ly6C^high^ Ly6G^−^) isolation using anti-Gr1 beads. The purity of isolated cells was > 95% for T cells and between 75 and 90% for MDSC.

Cells from bone morrow cultures were isolated by FACSorting. Cells were stained as described for FACS analysis. FVD^neg^Gr1^high^MHC-II^low^ and FVD^neg^Gr1^low^MHC-II^high^ were sorted on a BD Aria III system (BD Bioscience, Heidelberg, Germany).

### FACS analysis

Prior to fluorochrome staining of single cell suspensions, FcR III/II blocking was performed using the anti-CD16/CD32 TrueStain fcX™ antibody (BioLegend, London, UK). Dead cells were stained for exclusion with fixable viability dye (FVD) (Thermo Fisher Scientific, Karlsruhe, Germany). For cell-specific surface staining, cells were labeled with CD4 (clone GK1.5), CD8 (clone 53–6.7), CD11b (clone M1/70), CD11c (clone N418), CD45 (clone 30-F11), Gr1 (clone RB6-8C5), Ly6C (clone HK1.4), Ly6G (clone 1A8), MHC-II (clone AF6-120.1; all BioLegend, London, UK). IRF4 (clone IRF4.3E4; BioLegend, London UK) or isotype control (clone RTK2071; both BioLegend, London, UK) were stained intracellularly using the one-step intracellular staining protocol of the eBioscience™ FoxP3/Transcription Factor Staining Buffer Kit (Thermo Fisher Scientific, Karlsruhe, Germany). Samples were acquired on a BD LSRFortessa system (BD Bioscience, Heidelberg, Germany), and data were analyzed with FlowJo X software (FLOWJO LLC, Ashland, OR, USA).

### T cell suppression assay

Isolated T cells were stained with 2.5 µM Carboxyfluorescein succinimidyl ester (CFSE; Thermo Fisher Scientific) in PBS for 4 min at room temperature and reaction was stopped with FCS. For the assessment of MDSC suppressive capacity, MDSC were co-cultured with anti-CD3/anti-CD28 stimulated CFSE-labeled T cells. For this, 5 × 10^4^ T cells (per well) were seeded into 96-well plates and cocultured with 0.31 × 10^4^, 0.63 × 10^4^, 1.25 × 10^4^, 2.5 × 10^4^ or 5 × 10^4^ MDSC. Each well was supplemented with 1 µl beads (Dynabeads™ Mouse T-Activator CD3/CD28, gibco®, Thermo Fisher Scientific, Karlsruhe, Germany). After 72 h, CFSE dilution of CD4^+^ and CD8^+^ T cells was analyzed by flow cytometry (BD Canto II system, BD Bioscience, Heidelberg, Germany). Unstimulated CFSE-labeled T cells only were used to set the threshold of proliferated T cells (CFSE^low^).

### Human dataset analysis

Survival data, IRF4 expression level as well as information on metastasis status of PDAC patients from the TCGA cohort were retrieved via the Xena browser [37]. Two groups of patients with metastasized PDAC were analyzed: Patients with low IRF4 expression level (≤ the lower quartile) and patients with high IRF4 expression level (≥ the upper quartile). The survival of these two groups was analyzed in a Kaplan–Meier analysis and compared with a Wilcoxon test. The survival status after one year was displayed in a contingency table. As all expected cell frequencies were above five, *χ*^2^ test was used to compare one-year survival of the two groups. All analysis on the TCGA data set was performed using IBM® SPSS® Statistics 25. Graphs were generated in Graphpad Prism 8.3.

### Histology

Tumors were embedded in Tissue-Tek® O.C.T.™ (Sakura Finetek GmbH, Staufen, Germany), rapidly frozen in liquid nitrogen and stored at − 80 °C. Prior to analysis, samples were thawed, once washed in PBS, fixed in ROTI^®^Histofix 4% paraformaldehyde (Roth, Karlsruhe, Germany) and subjected to automated routine histological tissue processing. After paraffin embedding, four µm thick whole tissue sections were stained using haematoxylin-eosine (HE) staining (haematoxylin: Waldeck, Münster, Germany; eosine: Sigma) in an automated tissue stainer (Tissue Tek Prisma, Sakura Finetek).

### Statistical analysis

Data represent individual mice and are displayed as mean with standard error of the mean (SEM). To test for statistically significant differences between two groups, student’s t test (for expression data only) or Mann–Whitney U test was used. To compare more than two groups, we applied Kruskal–Wallis test followed by Dunn’s multiple comparison post hoc tests between selected samples. To analyze differences in the survival, Mantel–Cox Logrank test was conducted. For the statistical analysis of suppression assays, only the difference between the conditions with the highest concentration of MDSC was analyzed using a Mann–Whitney U test. Statistical analysis of murine data was performed in Graphpad Prism 8.3.

## Electronic supplementary material

Below is the link to the electronic supplementary material.

Supplementary Figure S1:**Histology of T110299 tumors from wild-type and Irf4**^**-/-**^** mice displays no apparent morphologic differences**. Representative sections of orthotopic T110299 tumors (two tumors per genotype) stained with haematoxylin and eosin (H&E) are depicted. Scale bars indicates 100 μm. (PDF 56572 kb)

Supplementary file2 (PDF 81 kb)
