# Supplementary figures and images for: Systemic but not MDSC-specific IRF4 deficiency promotes an immunosuppressed tumor microenvironment in a murine pancreatic cancer model
Source: Cancer Immunol Immunother. 2020 May 24;69(10):2101–12. doi: 10.1007/s00262-020-02605-9 (PMC7511276; doi:10.1007/s00262-020-02605-9)

Supplementary Figure S1

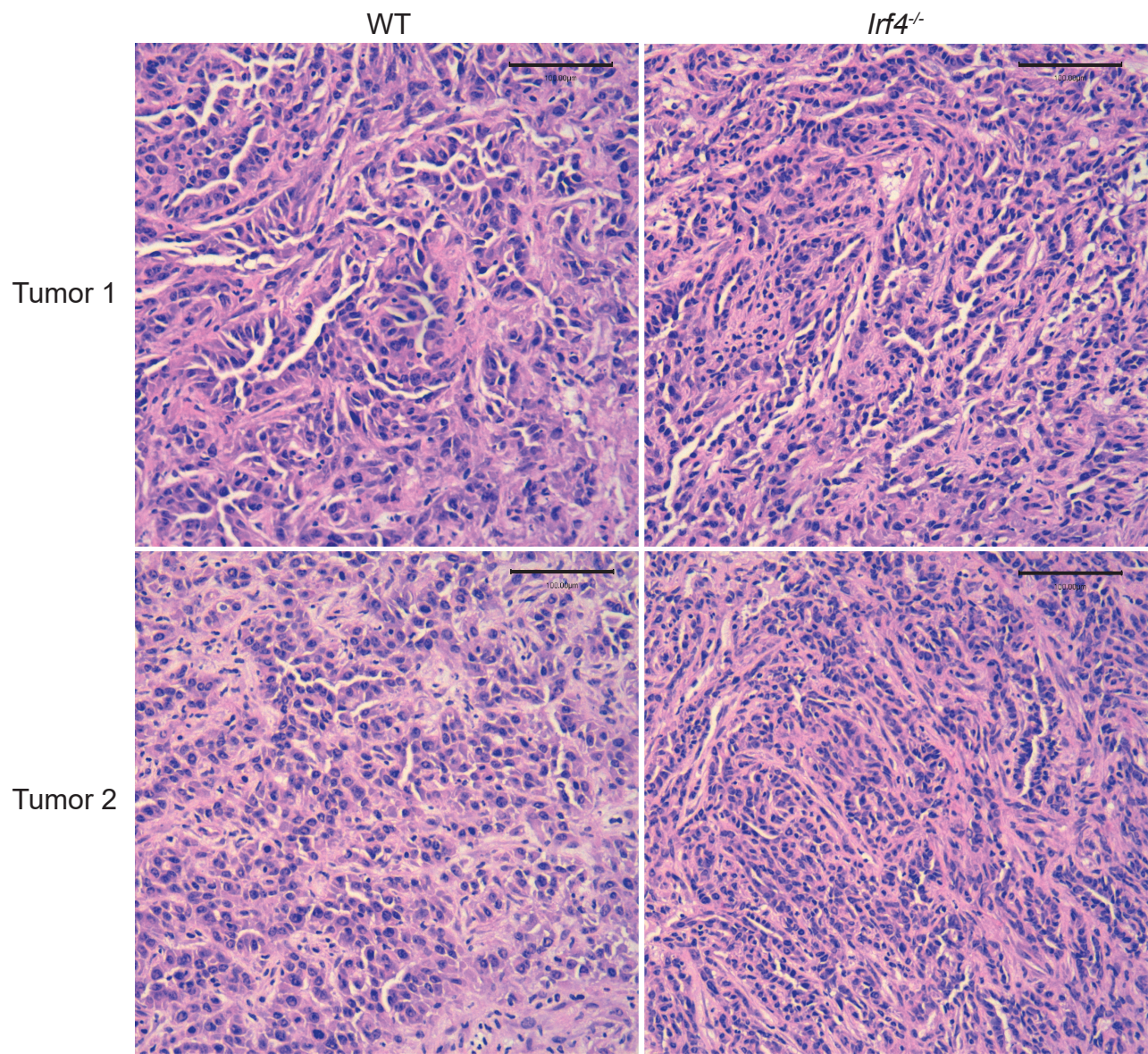

Supplement: Supplementary file 1 — Supplementary Figure S1:Histology of T110299 tumors from wild-type and Irf4-/- mice displays no apparent morphologic differences. Representative sections of orthotopic T110299 tumors (two tumors per genotype) stained with haematoxylin and eosin (H&E) are depicted. Scale bars indicates 100 μm. (PDF 56572 kb) [file 262_2020_2605_MOESM1_ESM.pdf]
