# Supplementary material for: Systemic but not MDSC-specific IRF4 deficiency promotes an immunosuppressed tumor microenvironment in a murine pancreatic cancer model
Source: Cancer Immunol Immunother. 2020 May 24;69(10):2101–12. doi: 10.1007/s00262-020-02605-9 (PMC7511276; doi:10.1007/s00262-020-02605-9)
Supplement: Supplementary file 2 — Supplementary file2 (PDF 81 kb) [file 262_2020_2605_MOESM2_ESM.pdf]

Supplementary Table 1

Name	Sequence 5`->3`
Ly6G_wt_ctrl_fwd	GAGACTCTGGCTACTCATCC
Ly6G_wt_ctrl_rev	CCTTCAGCAAGAGCTGGGGAC
Ly6G_Cre_1_fwd	GGTTTTATCTGTGCAGCCC
Ly6G_Cre_rev	GAGGTCCAAGAGACTTTCTGG
Ly6G_Cre_2_fwd	ACGTCCAGACACAGCATAGG
IRF4-fl_geno1_fw	TGGGCACCTCTACTGTCTGG
IRF4-fl_geno2_rv	CTCTGGGGACATCAGTCCT
IRF4-fl_geno3_rv	CGACCTGCAGCCAATAAGC
FLP_tg_geno1_fw	TGCCGGTCCTATTTACTCGT
FLP_tg_geno1_rv	TACTTCTTTAGCGCAAGGGGTAG
FLP_wt_geno1_fw	CTAGGCCACAGAATTGAAAGATCT
FLP_wt_geno1_rv	GTAGGTGGAAATTCTAGCATCATCC
LysM_geno1_fw	CCCAGAAATGCCAGATTACG
LysM_geno_rv	CTTGGGCTGCCAGAATTTCTC
LysM_geno2_fw	TTACAGTCGGCCAGGCTGAC
IRF4-wt-for-A2	TGCCTTTGGGACGGATGCTC
IRF4-wt-rev-B2	CTTCTAGCTGACCACTAAGAAC
IRF4-Δfrt-rev-D	AATCAAGTGTGGGCAAGACTG

Supplementary table 2

Genomic Locus	Initial Denaturation	Denaturation	Annealing	Elongation	Number of cycles	Final elongation
*IRF4^flox^*	94 °C, 5 min	94 °C, 30 sec	60 °C, 30 sec	72 °C, 30 sec	35	72 °C, 5 min
IRF4-/-	95 °C, 5 min	95 °C, 15 sec	62 °C, 30 sec	72 °C, 60 sec	30	72 °C, 5 min
*LysM^Cre^*	94 °C, 5 min	94 °C, 20 sec	60 °C, 15 sec	72 °C, 15 sec	25	72 °C, 2 min
*Ly6G^Cre^*	94 °C, 5 min	94 °C, 30 sec	60 °C, 30 sec	72 °C, 60 sec	35	72 °C, 10 min
*Ly6G^WT^*	94 °C, 5 min	94 °C, 30 sec	60 °C, 30 sec	72 °C, 60 sec	35	72 °C, 10 min
FLP1	94 °C, 5 min	94 °C, 20 sec	60 °C, 15 sec	72 °C, 15 sec	25	72 °C, 2 min
